# Ki67 proliferation in core biopsies versus surgical samples - a model for neo-adjuvant breast cancer studies

**DOI:** 10.1186/1471-2407-11-341

**Published:** 2011-08-07

**Authors:** Quinci Romero, Pär-Ola Bendahl, Marie Klintman, Niklas Loman, Christian Ingvar, Lisa Rydén, Carsten Rose, Dorthe Grabau, Signe Borgquist

**Affiliations:** 1 Department of Oncology, Skåne University Hospital, Lund, Sweden; 2 Department of Oncology, Clinical Sciences, Lund University, Lund, Sweden; 3 Department of Surgery, Skåne University Hospital, Lund, Sweden; 4 Department of Surgery, Clinical Sciences, Lund University, Lund, Sweden; 5 Department of Pathology, Skåne University Hospital, Lund, Sweden; 6 Department of Pathology, Laboratory Sciences, Lund University, Lund, Sweden

**Keywords:** core biopsy, Ki67, breast cancer, proliferation, neo-adjuvant

## Abstract

**Background:**

An increasing number of neo-adjuvant breast cancer studies are being conducted and a novel model for tumor biological studies, the "window-of-opportunity" model, has revealed several advantages. Change in tumor cell proliferation, estimated by Ki67-expression in pre-therapeutic core biopsies versus post-therapeutic surgical samples is often the primary end-point. The aim of the present study was to investigate potential differences in proliferation scores between core biopsies and surgical samples when patients have not received any intervening anti-cancer treatment. Also, a lack of consensus concerning Ki67 assessment may raise problems in the comparison of neo-adjuvant studies. Thus, the secondary aim was to present a novel model for Ki67 assessment.

**Methods:**

Fifty consecutive breast cancer cases with both a core biopsy and a surgical sample available, without intervening neo-adjuvant therapy, were collected and tumor proliferation (Ki67, MIB1 antibody) was assessed immunohistochemically. A theoretical model for the assessment of Ki67 was constructed based on sequential testing of the null hypothesis 20% Ki67-positive cells versus the two-sided alternative more or less than 20% positive cells..

**Results:**

Assessment of Ki67 in 200 tumor cells showed an absolute average proliferation difference of 3.9% between core biopsies and surgical samples (p = 0.046, paired t-test) with the core biopsies being the more proliferative sample type. A corresponding analysis on the log-scale showed the average relative decrease from the biopsy to the surgical specimen to be 19% (p = 0.063, paired t-test on the log-scale). The difference was significant when using the more robust Wilcoxon matched-pairs signed-ranks test (p = 0.029). After dichotomization at 20%, 12 of the 50 sample pairs had discrepant proliferation status, 10 showed high Ki67 in the core biopsy compared to two in the surgical specimen (p = 0.039, McNemar's test). None of the corresponding results for 1000 tumor cells were significant - average absolute difference 2.2% and geometric mean of the ratios 0.85 (p = 0.19 and p = 0.18, respectively, paired t-tests, p = 0.057, Wilcoxon's test) and an equal number of discordant cases after dichotomization. Comparing proliferation values for the initial 200 versus the final 800 cancer cells showed significant absolute differences for both core biopsies and surgical samples 5.3% and 3.2%, respectively (p < 0.0001, paired t-test).

**Conclusions:**

A significant difference between core biopsy and surgical sample proliferation values was observed despite no intervening therapy. Future neo-adjuvant breast cancer studies may have to take this into consideration.

## Introduction

Neo-adjuvant therapy is where a systemic cancer therapy is delivered with a therapeutic intention for a period of months prior to a local treatment for the primary tumor such as surgical removal [1-3]. In a pre-operative setting, where neo-adjuvant therapy is not yet recommended, a novel pharmaceutical can be evaluated in a so-called "window-of-opportunity" study with the experimental therapy given between diagnosis and tumor removal [4-10]. Comparison of the pre-treatment core biopsy and the post-treatment surgical sample establishes the efficacy of the experimental therapy often using change in tumor cell proliferation as a primary end-point [11-15]. Proliferation is a key feature of tumor progression and it is widely estimated immunohistochemically using the Ki67 antibody MIB-1. Ki67 is a nuclear protein of unclear function present in all proliferating cells, both normal and tumor [16,17]. Although widely used as a predictive marker in neo-adjuvant breast cancer studies, less is known about Ki67 expression in an untreated cohort and potential baseline disparity between core biopsies and their corresponding surgical samples [18,19]. Furthermore, there is a lack of consensus concerning the optimal number of cancer cells needed to achieve reliable Ki67 results [7,14,20-23]. The aim of this study was to map possible fundamental differences in Ki67 expression between core biopsies and their corresponding surgical samples. The secondary aim was to present a model for Ki67 assessment, which has the potential to make results from future neo-adjuvant studies more comparable.

## Materials and methods

A retrospective cohort of fifty consecutive breast cancer cases from 2008 and 2009, with both core biopsy and corresponding surgical sample available, were retrieved from the Department of Pathology, Skåne University Hospital, Lund, Sweden. No intervening anti-cancer treatment between the core biopsy sampling and operation had been given. The study was approved by the Ethical Committee at Lund University (Dnr 529).

### Histopathological analyses

According to common practice at the Department of Pathology, tumor specimens were formalin-fixed and representative parts of the breast carcinomas and all needle core biopsies were paraffin-embedded. Sections were cut at 4 μm, deparaffinized, and rehydrated in graded alcohols. Antigen retrieval was performed in a microwave oven in Citrate buffer pH 6 for 20 min. Expression of Ki67 was determined using the LSAB+, Dako REAL™ Detection Systems (K5001, Dako Glostrup, Denmark). The Ki67 antibody (clone MIB1 DAKO Glostrup, Denmark) was diluted 1:500 and incubated for 25 min in a TechMate 500 Plus (DAKO) and visualized with DAB (3,3'-Diaminobenzidine).

### Ki67 evaluation

First, eosin and hematoxylin stains were examined on 2× and 10× magnification to identify cancerous regions within a tissue sample. Second, the MIB-1 stain for Ki67 was examined on 2× and 10× magnification to identify hotspots, areas with increased numbers of Ki67 positive cells within the previously identified cancerous regions. Finally, using 40× magnification over the hotspot, 10 cancer cells at a time were evaluated. Nuclei more brown than blue were scored positive. The number of Ki67-positive tumor cells from each set of 10 was recorded. This procedure was performed 100 times for a total of 1000 cells. The field of magnification was divided visually into eight "pie slices" that were evaluated from the outer edge of the field towards the centre. The slices were evaluated in a clockwise order. When the entire field of magnification did not include 1000 cancer cells, a new field was chosen, often within the same hotspot and adjacent to the original field. Occasionally, the chosen hotspot did not contain 1000 cancer cells. In these cases the magnification was reduced and a new hotspot was located and evaluated in the manner described above. In cases with no additional hotspots, a random field of tumor cells was chosen and evaluated, until a count of 1000 was reached. In cases where no initial hotspot could be discerned, the fields were chosen at random and 1000 cancer cells were evaluated. Each core biopsy and surgical sample was evaluated twice, four weeks between evaluations, by a single observer (QR) with the observer blinded to the relationships between samples. Surgical samples were counted first in a random order followed by the core biopsy samples, also assessed in random order. The observer (QR) is a medical doctor and a PhD student at the Department of Oncology/Pathology and has undergone supervised education in Ki67 assessment by a senior breast pathologist (DG).

### Statistical analysis and model development

**Figure 1:**
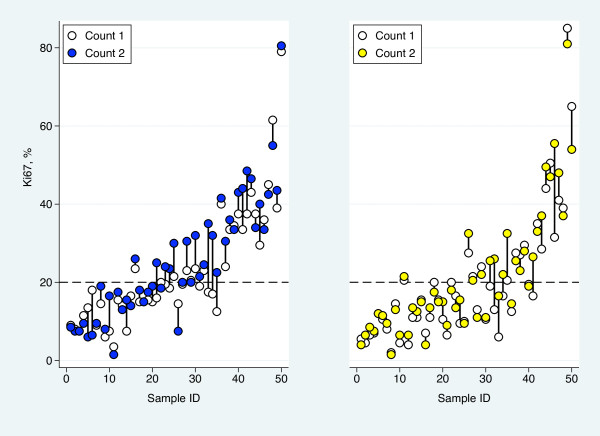
**Comparison of Ki67 results**. Variation in Ki67-determined proliferation values for core-biopsies (left) and surgical samples (right) between counts 1 and 2 of 200 tumor cells.

**Figure 2:**
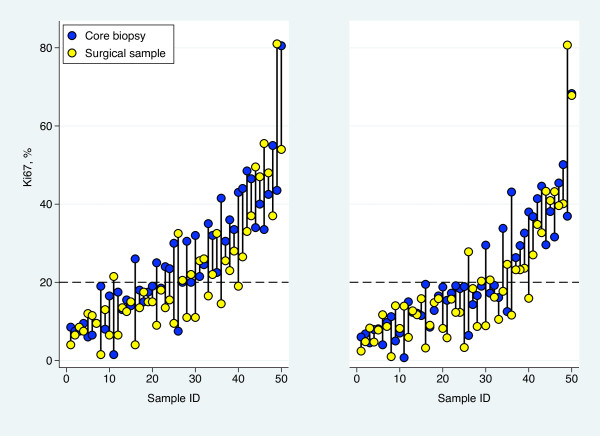
**Differences in proliferation**. Proliferation values for core biopsies and surgical samples as determined using the Ki67 bio-marker for the 200 (left) and 1000 (right) tumor cells evaluated.

**Figure 3:**
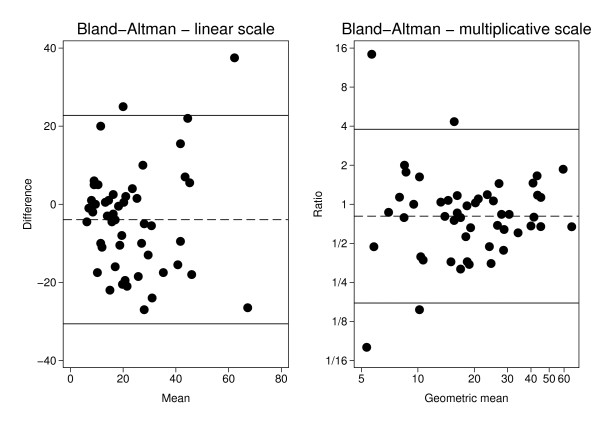
**Bland-Altman plots for Ki67-positive cells in surgical samples and core biopsies**. (3a) The difference in percentage of Ki67-positive cells between surgical sample and core biopsy versus the corresponding mean for each of the 50 pairs. Two hundred cells were counted. The dashed line represents the average difference 3.9% and the solid lines a 95% reference interval around this estimate. (3b) The ratio of the percentage of Ki67-positive cells in the surgical sample to the corresponding percentage in the core biopsy versus the geometrical mean for each of the 50 pairs. Two hundred cells were counted. The dashed line at 0.81 represents the geometric mean of the ratios (19% lower Ki67 fraction the surgical sample) and the solid lines a 95% reference interval around this estimate.

Differences in Ki67 levels between core biopsies and surgical samples were in a pre-planned manner evaluated for 200 and 1000 tumor cells, based on the number of tumor cells evaluated in Swedish clinical practice and the number of tumor cells commonly counted in breast cancer studies [7,14,20-23]. Ki67 differences with and without log-transformation of the values, were evaluated using the paired t-test. Analysis on the original scale corresponds to comparison of absolute percentages whereas analysis on the log-scale compares relative percentages. The paired t-test was also used to compare Ki67 levels within sample types, comparing the initial 200 to the final 800 cells counted. Since outliers were detected, the null hypothesis of equal distributions was also tested non-parametrically using the Wilcoxon matched-pairs signed-ranks test. Bland-Altman analysis was used to assess heteroscedasticity and trends in bias with increasing Ki67 value. The order of the samples (Sample ID in Figures 1, 2 and 3) is from lowest to highest proliferation based on the average over core biopsy and surgical sample for 200 counted cells.

A dichotomized Ki67 variable was created using 20% positive cells as cut-off. The McNemar test was used to evaluate if differences in both directions were equally likely after dichotomization. The cut-off value of 20% was chosen based on the present data, classifying one-third of the samples as positive and two-thirds as negative, and on previous Swedish studies indicating that a cut-off near the seventh decile correlates with the highest risk of developing metastases [24]. Model development commenced by defining a stopping rule for the number of cells necessary to count in order to reject the null hypothesis of 20% Ki67-positive cells. The rule was based on the assumption of a homogeneous Ki67expression. A sequential test strategy was adopted, which allows testing of the null hypothesis. Samples are first evaluated by counting cancer cells in ten cell increments and recording the number of Ki67-positive cells. Testing of the null hypothesis begin when 100 cells have been evaluated. Thereafter testing is done at 10 cell cumulative intervals i.e. 110, 120 and up to 400 counted cancer cells. An exact two-sided binomial test was used and the significance level was set to 0.012 for each test. The rationale behind this nominal p-value is that simulation studies show that it preserves the overall significance level of the procedure at approximately 0.05.

All tests were two-sided and the significance level was set to 0.05. The statistics package Stata 11.1 (StataCorp LP, College Station, TX, USA, 2010) was used for statistical analysis.

## Results

### Patient and tumor data

Among the fifty breast cancer cases four women had previously had breast cancer in the contra-lateral breast and one woman had recurrent breast cancer in the ipsi-lateral breast. Mean time interval from core biopsy sampling to surgery was 31 days with a median of 28 days and a range from 8 to 77 days. Nodal status was assessed according to AJCC 6^th ^[25] with one third of the patients being node positive. Further patient and tumor characteristics are presented in Table 1.

**Table 1:** Distribution of clinicopathological variables and immunohistochemical markers

		n (%)
**Age: median (range)**	**63 (25-88) years**	

Primary breast cancer	Yes	49 (98)
	No*	1 (2)
Tumor size	<20 mm	30 (60)
	≥20 and <50 mm	16 (32)
	≥50 mm	4 (8)
Tumor type	Ductal	44 (88)
	Lobular	5 (10)
	Mucinous	1 (2)
Histological grade	1	16 (32)
	2	19 (38)
	3	15 (30)
Estrogen receptor status**	Positive	45 (90)
	Negative	4 (10)
Progesterone receptor status**	Positive	37 (74)
	Negative	13 (26)
HER2 status***	Normal	32 (64)
	Amplified	8 (16)
	Unknown	10 (20)
Axillary node Status†	Positive	19 (38)
	Negative	30 (60)
	Unknown	1 (20)

* Four women had contralateral primary tumors and one woman had recurrent breast cancer in the ipsilateral breast.

** Positive estrogen and progesterone receptor status when ≥ 10%

*** Patients ≥ 70 years of age and surgery before May 2008 did not routinely receive an HER2 test.

† See reference given in text.

### Agreement between two repeated Ki67 assessments

Ki67 assessment of each sample pair, core biopsy with corresponding surgical sample, was performed twice. The mean difference of the two evaluations of the 50 core biopsies at 200 cells level was 2.6% and the standard deviation of the differences 5.7%. The corresponding figures for the surgical samples were 1.8% and 5.6%, respectively (Figure 1). For both sample types, the average was significantly higher in the second evaluation (p = 0.002 for core biopsies and p = 0.02 for surgical samples) but the general shift of level was not significantly different for the two sample types (p = 0.44). Prior to the above described analyses of potential differences between the two counts the second count was randomly chosen and used for all statistical analyses.

### Ki67 assessment of 200 cancer cells

The first 200 of the 1000 cancer cells evaluated for Ki67 were analyzed separately based on Swedish clinical practice and the experience of the first 200 tumor cells counted in the vast majority represent a single hotspot. Core biopsies showed a mean proliferation 3.9% higher than the surgical samples' mean proliferation (95% CI: 0.1-7.8%, p = 0.046, paired t-test), but no consistent pattern of either the core biopsy or surgical sample being higher than the other was seen (Figure 2). Sensitivity analysis revealed one pair of samples to be highly influential. When excluding this sample pair, with an extreme difference in Ki67, the mean difference increased to 4.8% (95% CI: 1.2-8.3%, p = 0.009). This profound effect of a single sample pair on the results suggested that the assumptions behind the t-test for the complete set of 50 tumors are questionable. The null hypothesis of equal distributions was, however, also rejected when using the non-parametric Wilcoxon matched-pairs signed-ranks test (p = 0.029). The median absolute difference between core biopsies and the corresponding surgical sample was 3.5% with a range from -38% to +27%. Analysis on the log-scale showed the Ki67 values from the surgical specimens to be on average 19% lower (geometric mean of the ratios = 0.81, 95% CI: 0.65-1.01) compared to the values from the core biopsies (p = 0.063). Also this estimate is influenced by extreme values, as can be seen in the Bland-Altman plots for the two scale alternatives presented in Figure 3. On the linear scale, the variability seems to increase with increasing Ki67-level whereas the opposite pattern is seen on the multiplicative scale. The bias does not seem to vary with increasing Ki67-level.

Dichotomization of Ki67 into categories of high or low proliferation using a 20% cut-off value resulted in 12 sample pairs with discrepant Ki67 status. Ten patients had core biopsies classified as highly proliferative and their respective surgical samples classified as low proliferation, while two patients showed the opposite pattern (Table 2). This disparity in classification demonstrated significant skewing (p = 0.039, McNemar's test). All statistical methods employed show core biopsies as consistently and significantly more proliferative than their corresponding surgical samples despite no intervening treatment. Table 3 summarizes these results.

**Table 2:** Dichotomization of core biopsies and surgical samples into low and high proliferation as determined by Ki67 evaluation using a 20% cut-off value

	200 cells			1000 cells		
	**Core biopsies**					

Surgical samples	< 20%	>20%	Total	< 20%	> 20%	Total

< 20%	19	10	29	29	4	33

>20%	2	19	21	4	13	17

Total	21	29	50	33	17	50

**Table 3:** Summary of statistical analysis for 200 and 1000 cancer cells after evaluation for Ki-67 proliferation

Statistical test	200 cells		1000 cells	
	
	Mean difference (95% CI)	p	Mean difference (95% CI)	p
t-test, linear scale	3.9% * 0.1% - 7.8%	0.046	2.2%* -1.1% - 5.6%	0.19

t-test, linear scale excluding outlier	4.8% * 1.2% - 8.3%	0.009	3.1%* 0.03% - 6.0%	0.03

t-test multiplicative scale	0.81 ** 0.65-1.01	0.063	0.85 ** 0.67-1.08	0.18

Wilcoxon***		0.03		0.06

	Disparate pairs (distribution)	p	Disparate pairs (distribution)	p

Dichotomy McNemar's test	12 (10+2)****	0.04	8 (4+4)****	1.0

*Core biopsies' average proliferation is higher than surgical samples'

** Geometric mean of the ratios (surgical specimens/core biopsies)

*** Wilcoxon matched-pairs signed-ranks test

****Instance of core biopsies as more proliferative + instances of surgical samples as more proliferative

### Ki67 assessment of 1000 cancer cells

Analysis of 1000 cancer cells evaluated for Ki67 proliferation showed core biopsies having an absolute mean proliferation value 2.2% higher than the surgical samples (p = 0.19, paired t-test). Excluding the single outlier pair increased the mean difference to 3.1% (p = 0.030). The median difference in proliferation values between core biopsies and surgical samples was 2.5% with a range from -44% to +32% - a difference which was not significant (p = 0.057) when tested non-parametrically using the Wilcoxon matched-pairs signed-ranks test. The geometric mean of the Ki67 ratios (surgical sample/core biopsy) was 0.85 corresponding to an average relative decrease of 15% (p = 0.18, paired t-test on the log-scale).

Dichotomization into categories of high or low using the 20% cut off-value resulted in eight discrepant pairs with four patients having core biopsies classified as highly proliferative and respective surgical samples classified as low proliferation, and another four patients with the opposite pattern. The disparity in classification demonstrated no skewing (p = 1.0, McNemar's test). None of the statistical methods employed show a significant difference between core biopsy and corresponding surgical sample proliferation values excepting the t-test excluding the outlier (Table 3).

### Ki67 evaluation accuracy

**Figure 4:**
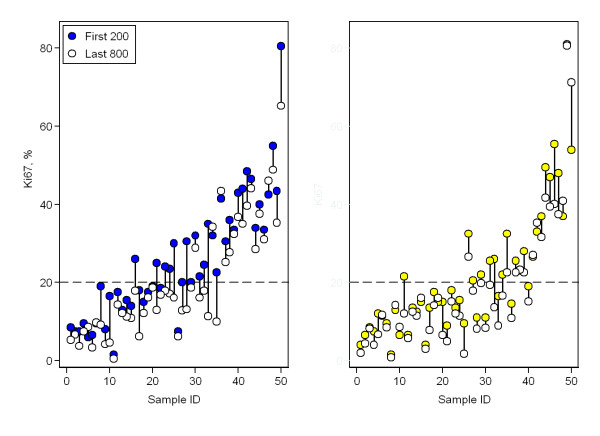
**The dilution effect**. Comparison of Ki67-determined proliferation values of core biopsies (left) and surgical samples (right) from the initial 200 versus the final 800 tumor cells evaluated.

**Figure 5:**
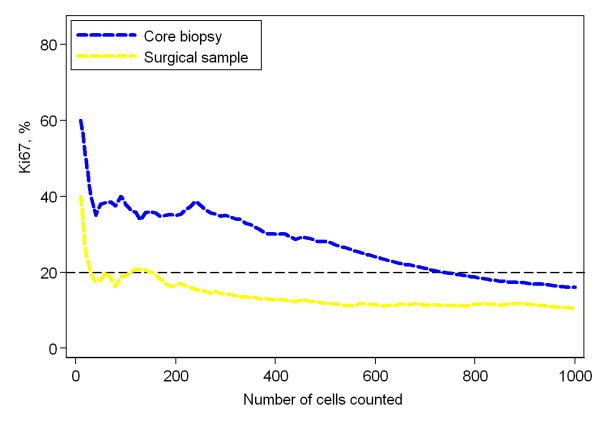
**Single case demonstration of the dilution effect**. Cumulative proliferation values for both core biopsy and surgical sample as determined using the Ki67 bio-marker from a single patient case. Shown here is a cumulative proliferation value near the pre-determined cut off-value of 20% changing classification of the sample from high- to low proliferative after inclusion of cells outside a hotspot.

**Figure 6:**
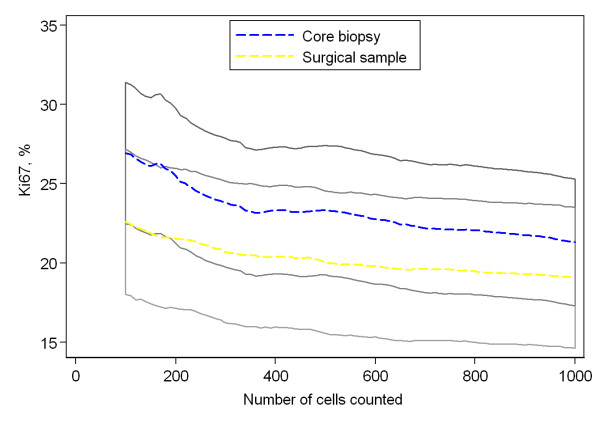
**The mean dilution effect**. For each sample type, the mean percentage of Ki67-positive cells, over the 50 samples, is plotted against the number of cells counted. The dashed blue line corresponds to core biopsies and the dashed yellow line to surgical specimens. The shaded areas represent pointwise 95% confidence intervals for the means. The confidence bands are overlapping. Therefore, an intermediate grayscale was used for the overlapping area. Please, note that the y-scale does not start at 0%.

Of the 1000 cancer cells evaluated for Ki67 proliferation, the initial 200 cells were compared with the final 800 in a broad attempt to visualize a suspected dilution effect (Figure 4). The mean proliferation value among core biopsies was 25.5% and 20.2% for the initial 200 and final 800 tumor cells, respectively, an absolute difference of 5.3%. The mean proliferation value among surgical samples was 21.6% and 18.4% for 200 and 800 cells respectively, an absolute difference of 3.2%. Both differences were significant according to the paired t-test, p < 0.0001. Figure 5 is illustrative of this dilution effect and its plausible consequence when a sample's proliferation value is near the cut off-value. The dilution effect can also be seen in Figure 6, where the mean proliferation estimate over all the 50 samples of each type is plotted against the number of cells counted.

### Theoretical model for Ki67 evaluation

**Figure 7:**
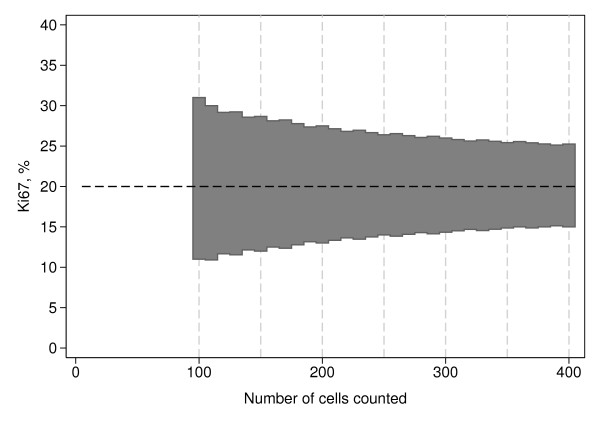
**A theoretical model for Ki67 evaluation**. The shaded area represents the fractions for which the null hypothesis of 20% Ki67-positive cells could not be rejected when tested sequentially after 100, 110, 120, ..., up to 400 evaluated cells.

Assuming homogeneity of the sections with regard to Ki67 staining and a fixed number of *n *counted cells, it is straightforward to calculate an exact *k*% confidence interval (CI), based on the binomial distribution, for the proportion of Ki67-positive cells under the null hypothesis of 20% positivity. An observed proportion outside this interval means that the null hypothesis can be rejected at the (100-k)% significance level. We suggest to test the null hypothesis not only for one *n*, but sequentially starting with *n *= 100 and proceeding in steps of 10 cells until either significance or a maximum of 400 counted cells. The lower limit was chosen to get a reasonably stable estimate of the proportion of Ki67-positive cells and the upper limit for practical reasons and to avoid excessive dilution effect. Twenty samples of the 100 included in our series require evaluation of more than 400 tumor cells to reliably determine Ki67-status. This combined with an uncertainty as to when the dilution effect begins to affect results encouraged the conservative maximum of 400 cells for evaluation. Extensive simulations under the null hypothesis showed that *k *= 98.8 preserves the overall significance of the sequential test procedure at 5%. The corresponding rejection regions can be found in Figure 7.

## Discussion

Biological tumor markers are used as measures of clinical efficacy when evaluating novel neo-adjuvant therapies., Ki67 is a biological tumor marker that follows changes in tumor proliferation between pre- and post-therapeutical samples, typically core biopsies and surgical samples [10,26,27]. These, however, vary in both sample acquisition and post-acquisition treatment while containing compositional differences possibly affecting direct comparison [28]. To the best of our knowledge these potential differences have not previously been fully addressed [18,19,29,30]. In this study, we observe significant average proliferation differences between paired core biopsies and surgical samples from patients in an untreated setting. Importantly, the difference represents an average difference in proliferation with the core biopsies demonstrating a higher proliferation index compared to the surgical samples. The pattern is however inconsistent between individuals with proliferation differences in either directions as demonstrated in Figure 2 and 3. This variability needs to be addressed in interpretations of proliferation differences in future clinical studies. In many cases a decrease in proliferation values with increasing number of evaluated tumor cells was observed. This dilution effect, which we believe to affect core biopsies and surgical samples unequally, could also play a role in the systematic difference observed. Finally, the lack of consensus concerning Ki67 assessment may raise problems in the comparison of neo-adjuvant studies. We propose a theoretical model for Ki67 assessment which may diminish the reduction of systematic differences and improve comparison of future neo-adjuvant studies.

The decision of including fifty tumors in this study was not based on a statistical rationale or availability of tumor tissue, rather regarded as a reasonable number of samples for detailed Ki67 assessments in this preliminary study.

Initial analysis using the t-test showed a significant difference between average proliferation values of core biopsies versus surgical samples for the first 200 cancer cells counted, but not for the entire 1000 cells. However, when a single sample pair with an extreme difference in proliferation was excluded, significant differences were observed for both 200 and 1000 cancer cells. This profound effect of a single sample pair suggests that the distributional assumptions of the t-test are not met. Hence, a non-parametric analysis, using Wilcoxon matched-pairs signed-ranks test, was carried out for the entire series of 50 sample pairs. This analysis revealed a significant difference in Ki67 expression between core biopsies and surgical samples, but only for the first 200 tumor cells counted. The Ki67-fractions in core biopsies and surgical samples were also compared on a multiplicative scale - i.e. as ratios. This scale should theoretically be a better choice, compared to the linear, if the variation in Ki67 increases with increasing average value, but the drawback is that ratios for low Ki67 values are unreliable. The geometrical mean of the ratios (surgical sample/core biopsy) was 0.81. Thus, the decrease in the surgical specimen was, on average, 19% relative to the core biopsy (p = 0.063).

The observed differences in Ki67 expression between the two types of samples leads to discordances after dichotomization at 20% and, for 200 cells, positivity in the core biopsy and negativity in the surgical sample was significantly more common than the opposite pattern. Random differences between the paired samples were expected due to the heterogeneity of the samples, but the significant systematic difference of the first 200 cells was unexpected. A closer examination of the statistical results was undertaken to better penetrate probable causes.

For clinical use, a dichotomized biomarker value is often preferred as a decision-making tool in choice of therapy. A dichotomized proliferation variable was thus created and tested statistically with results summarized in Table 2. At first glance, evaluating 1000 cancer cells results in fewer sample pairs with opposing classifications while eliminating the systematic difference seen with 200 cells, however, some cumulative Ki67 curves (Figure 5) demonstrate a slight but visible inverse relationship between the number of tumor cells evaluated and proliferation results, a dilution effect. When a sample's proliferation value is near the cut-off value and cancer cells outside of the initial hotspot are included in the assessment, researchers run the risk of diluting the actual proliferation percentage from one categorized as highly proliferative to one that is falsely classified as low proliferating. To verify this putative dilution effect, proliferation values based on the initial 200 cells were compared with the proliferation values of the latter 800 cells. Values for the first 200 cells were significantly higher in both core biopsies and surgical samples despite the evaluations coming from the same samples. Following this substantiation of the dilution effect we postulate sample composition and acquirement as primary factors which are discussed below.

Ki67 evaluation focused on hotspots. In nearly all samples the initial area of increased proliferation, or hot-spot, was exhausted before 1000 cells were evaluated leading to areas of lower proliferation being included in the final Ki67 result. Core biopsies generally contain fewer cancer cells than surgical samples. Studies suggest core biopsies are often acquired from near the center of a tumor, although knowledge of which area of the tumor the needle targets, is difficult to elucidate and might be regarded as random [31,32]. Hotspots, however, are often noted to occur near the periphery of a tumor [20]. Therefore, core biopsies could be expected to have lower proliferation values; however, core biopsy samples must pass through the tumor periphery in order to reach the tumor center and thus may pass through a hotspot. Assuming a core biopsy includes a hotspot and at the same time contains fewer cells than a surgical sample, the hotspot in the core biopsy would be less affected by dilution than a surgical sample containing not only entire hotspots, but large areas of low proliferation. Further explanations for the observed systematic difference relate to both acquisition and post-acquisition handling of tissue samples. Acquisition of core biopsies is a relatively quick process with little time for ischemic damage to affect the sample. Surgical samples are, however, routinely exposed to varying periods of ischemia during tumor removal. This hypoxic damage could result in apoptosis of surgical sample cancer cells and lead to lower proliferation values compared to core biopsies lacking significant hypoxic damage. Post-acquisition handling of tissue samples also varies between core biopsies and surgical samples. Core biopsies are immediately fixed in formalin while surgical samples are often stored on ice for varying lengths of time before commencing formalin fixation. Cold ischemic damage could lead to further apoptosis. The nearly instantaneous acquisition and fixation of the core biopsies allows not only minimal time for apoptosis, but little opportunity for degradation of the Ki67 nuclear protein, whereas the combined ischemic times during and after surgery give ample opportunity for protein degradation to occur [11]. Further studies are required to elucidate the extent to which these factors influence the observed difference between core biopsies and surgical samples.

The dilution effect, regardless of cause, might be important to note in clinical pratice if samples are dichotomized, as only proliferation values near a chosen cut-off would be affected by the dilution effect. In a research context, however, where continuous values are gathered and analyzed as such, the dilution effect could be relevant over the majority of samples.

A lack of consensus concerning an appropriate cut-off value for Ki67 exists within the breast cancer research community [33-36] and might raise problems in comparison of neo-adjuvant studies using change in proliferation as an endpoint. The secondary aim of the present study was to introduce a theoretical model for Ki67 assessment which may also minimize the difference in proliferation observed here between core biopsies and surgical samples. The initial idea of a simple adjustment factor was discarded due to large ranges and intra-patient proliferation differences in both directions (Figure 2 and 3). Instead, we focused on the development of a theoretical model both optimizing the number of cancer cells evaluated for Ki67 and possibly standardizing the counting practice.

Currently, a predetermined number of cancer cells are evaluated without regard to sample heterogeneity and without a general agreement as to an optimal number. It is generally assumed that more cells evaluated signifies more reliable results as attested to by narrower confidence intervals. The underlying assumption when constructing a CI for the probability of Ki67-positivity, however, is that the counted cells constitute a random sample of cells from a homogenous distribution - an assumption which is certainly not true for small hotspots. We observed a dilution effect that despite narrower CIs provides less accurate Ki67-estimates for samples with small hotspots (Figures 4, 5 and 6). Optimally, cancer cells from a single hotspot are counted until the null hypothesis of proliferation rate equal to the cut-off can be rejected at a pre-defined significance level. Hotspots, however, vary considerably in size and composition from sample to sample. A dual problem of accommodating individual sample heterogeneity while optimizing counting methods emerges.

In summary, we propose the following counting model to be tested in future neo-adjuvant studies: Evaluate 100 cells for Ki67 proliferation. If the proportion is far enough from the cut-off value then no further cells need to be counted. If the cut-off cannot be excluded, an additional ten cells are evaluated and the corresponding proportion is compared to the limits in Figure 7. The evaluation continues in ten cell increments until either the cut-off is rejected or until a maximum of 400 cells is reached. In the latter case, the sample is designated unclassifiable.

## Conclusions

We find it superfluous, even detrimental, to evaluate 1000 cancer cells for Ki67-based proliferation. The observed inverse relationship between the number of tumor cells evaluated and proliferation results, is interpreted as a dilution effect. A significant difference between core biopsy and surgical sample proliferation values was observed for 200 cancer cells despite no intervening therapy. We postulate sample acquisition, post-acquisition treatment and compositional differences, in addition to the demonstrated dilution effect, between core biopsies and surgical samples to play roles. We propose a theoretical model for cell count optimization that hopefully contributes to a reduction of systematic differences while standardizing the counting practice for the comparison of future neo-adjuvant studies. However, this retrospective study requires further validation in an independent set of samples. Furthermore, studies focusing on sample handling and Ki67 degradation plus additional refinement of the theoretical model are of interest.

## List of abbreviations

DAB: 3,3'-Diaminobenzidine; CI: confidence interval.

## Competing interests

The authors declare that they have no competing interests.

## Authors' contributions

QR, PB, DG, and SB conceived and designed the study. QR and DG performed the pathological assessments. PO performed data analysis, QR and SB wrote the manuscript with the assistance of PO and DG and contributions from MK, LR, NL, CI, and CR, All authors approved the final manuscript.

## Pre-publication history

The pre-publication history for this paper can be accessed here:

http://www.biomedcentral.com/1471-2407/11/341/prepub
